# Paying Patients in General Hospitals

**Published:** 1895-03-02

**Authors:** 


### The Institutional Workshop.

### HOSPITAL ADMINISTRATION.

### PAYING PATIENTS IN GENERAL HOSPITALS.

The meeting of the governors of the Great Northern Central Hospital on the 22nd ult., a full report of which will be found on another page, marks a new departure in the history of the administration of metropolitan hospitals. So long ago as 1882, Mr. Burdett was invited by a local committee, including several medical men, to give an address at the Athenæum, Camden Road, on "How to Provide a Hospital for North London." In the course of that address, which was delivered at a large meeting on April 26th, 1882, it was shown amongst other things:

1. That at that time there was only one hospital bed in the district for every 33,000 of the inhabitants.

2. That cases had occurred where death had been hastened if not caused by the distance which patients resident in North London had to traverse in order to secure hospital treatment.

3. That a new general hospital in North London was likely to be financially successful, for reasons stated, which have proved, as will be seen from the report of the meeting, to be abundantly justified, by the funds which have flowed into the coffers of the Great Northern Central Hospital.

4. That providing care was taken not to add one more to the pauperising agencies of this vast metropolis, and that its constitution provided for the free relief of the poor, whilst it guarded the recipients of its benefits against improvidence and loss of self-respect, there was an opportunity in North London not only to do much good, but to do it in the right way, by establishing the Great Northern Central Hospital. In a word, the hospital must be conducted in a liberal but provident spirit, and the following plan for the admission of patients was suggested: "The in-patient department to consist of free and pay beds and paying wards, and the regulations for admission to be somewhat as follows: The burden of proof must rest on the patient. In other words, he will be called upon to show to the satisfaction of the committee that he is unable to pay anything, or only a certain sum per week for his maintenance when in hospital. When a patient is brought to the hospital, except in cases of accident, which will be admitted at once, the resident medical officer will decide what amount the applicant is to pay for board, such a rate being not less than the lowest amount prescribed by the committee. If the funds of a patient become exhausted before he is completely recovered from his illness, he may at the committee's discretion be transferred to a free bed. The committee to have power to consider from time to time the circumstances of any patient, and to alter the terms on which he has been received . . . . . Patients to be provided for by the establishment of private wards for pay patients only. Possibly, as in America, some of these private wards will be endowed and specially furnished at the cost of the representatives of churches, schools, and other similar bodies. In times of exceptional trade depression, an epidemic of sickness, or when unusual casualties occur, it may be found desirable to suspend the regulations with respect to pay patients, to enable the hospital authorities to meet the increased demand which at such a time will not unnaturally be made for free medical relief."

5. That a portion of the patients' payments should be set aside for the remuneration of the medical officers. The medical profession has already done too much gratuitous service in this country, and in any new departure this point should have due consideration. It will be the more easy in this instance, because all the medical agencies in the district could be brought into working relations, and the medical officers should be appointed mainly, if not entirely, from amongst the members of the profession who reside in North London.

#### Early Principles and Organisation.

As a result of Mr. Burdett's address, much interest was excited throughout North London, and it was determined to establish a general hospital which should "combine the pay with the free system," and provide that "those in-patients who paid might be treated by medical men of their own selection." It was further determined that the out-patients' department should be so regulated as to ensure that those only should receive gratuitous treatment who were fit objects for charity. A great public meeting was held at the Highbury Athenæum, April 28th, 1883, the Duke of Westminster in the chair, when it was moved by the late Professor Leoni Levi, seconded by Mr. Burdett, and resolved, "That recognising the advantage of combining the free with the graduated pay system, and of representative administration, this meeting resolves to establish a hospital on these principles, to be called a Central Hospital for North London." An immediate result of this meeting was the reception of overtures from the authorities of the Great Northern Hospital, situated in the Caledonian Road, which resulted in an amalgamation of forces in 1884, and the combined hospital was known thenceforward as the Great Northern Central Hospital. The chairman of the Great Northern Hospital, Mr. C. T. Murdoch, became the chairman of the new committee, and Mr. D. Marshall Lang, the chairman of the executive committee of the proposed new hospital, became deputy-chairman to the committee of the combined scheme.

#### The Site, Architects, and Constitution.

Steps were promptly taken to secure a site, the chief burden of this work being borne by Mr. Lang, Mr. Burdett, Mr. Black, and others who had been particularly identified with the first meeting in 1882. A limited competition among the most experienced hospital architects was organised, and plans were prepared, Mr. Burdett being requested as an expert to report upon them and assist the committee in making a selection. In the end, the plans of Messrs. Young and Hall were chosen, and the present site secured, with the result that a magnificent hospital, combining the rectangular and circular system of wards, with a separate wing for pay patients, has been built. The governors met in the board-room of the new hospital for the first time on the 22nd ult. Such is briefly the early history of the Great Northern Central Hospital, and it is necessary to remember the facts here stated in order to properly understand the proceedings at the annual meeting on Friday week. From the outset of the proposed new hospital it was made perfectly clear to everybody in and out of the district that the constitution was to combine the free and pay systems of administration, and to secure a new departure in hospital relief by preventing hospital abuse and the pauperisation of those who might resort to the hospital.

#### What the Money was Subscribed to Accomplish.

It must be remembered that all the money for the new hospital was given in support of the combined system of free and pay beds. How popular this scheme was in Islington and elsewhere is shown by the circumstance that there has been a magnificent growth in the contributions to the Great Northern Central Hospital under every head. Thus, since 1882 upwards of £55,000 had been given to the building fund. The ordinary income of the hospital, which in 1882 was but £3,686, has increased to £7,859 in 1894. The invested funds, which in 1882 amounted altogether to £13,028, increased to £20,968 in 1894. And after the large sale of stock (upwards of £10,000) referred to by the chairman, and required to wipe off the building debt, there remains a sum invested which exceeds £14,000, *i.e.*, more than the whole invested property in 1882. The ladies of Islington have raised by their unaided exertions no less a sum than £15,000, a fact which confers the greatest credit upon them, and they were worthily represented at the governors' meeting by two or three of their number who spoke with a pathos and force which won all hearts. There can be no question, therefore, that the combined pay and free system is popular in Islington and London generally, as that system was put forward at public meetings and by circular letters before a single stone of the present hospital was laid. The governors are therefore pledged to this plan of administration. It is part of the constitution of the Great Northern Central Hospital, and the governors could not with honour revert back to the old bad system of pauperising methods of medical relief. Besides, great and extra expenditure has been incurred to make the special wing in all respects as perfect as possible for the treatment of pay patients. It is too late, therefore, to go back now, and we are glad the governors so decided on the 22nd ult.

### The Admission of Patients.

At the outset it was thought to be undesirable to erect the whole of the hospital, and in consequence only the rectangular wing was built with the out-patients department, which it may be said, in passing, is one of the best in the country. The patients were transferred from the old building in the Caledonian Road to the new hospital buildings in 1888. So long as only a portion of the hospital was erected, the question of the admission of paying patients was left in abeyance, and all beds were free. Funds continued to flow in, and the committee soon felt themselves justified in giving out the whole of the contracts, and the entire hospital was completed and made ready for the reception of patients in the course of the year 1894. So soon as the administration block which contains the pay wards had been built, the question of the admission of paying patients had to be faced. Certain regulations were drawn up for the admission of paying patients, and advertisements were inserted in the newspapers inviting those who needed such accommodation to seek admission in due course, according to a fixed tariff. Directly the advertisements appeared an outcry arose amongst the general practitioners in North London, who assembled together in public meeting on more than one occasion, memorialised the committee of the hospital, and assumed an attitude of active opposition to the admission of pay patients. In the result, the committee undertook to advertise the pay wards no more, and agreed to modify the terms of admission, by limiting the payments to a maximum of two guineas a week per patient, and providing that all patients should be treated by the medical staff without charge. The following are the regulations at present in force:

I.—The pay wards are intended for the benefit of patients of limited incomes who are willing to contribute towards the cost of their maintenance when in the hospital.

II.—Except in urgent cases, patients can be admitted only after consideration of their applications by the House Committee of the hospital. The committee meets to receive applications every Wednesday at 5.30 p.m. Each application must be accompanied by a recommendation from a registered medical practitioner.

III.—Intending patients may obtain forms of application, and information as regards payments, from the secretary at the hospital.

IV.—No patient can be received without a guarantee in writing (on a form provided for the purpose) from some responsible person, for the necessary payments and for the removal of the patient when required.

V.—Patients will be admitted under the care of the medical officers of the hospital, who alone will be permitted to prescribe the treatment to be carried out in each case.

VI.—Patients may be visited by their friends as follows: Sundays, 1.30 to 3; Tuesdays and Fridays, 5 to 6. Permission to visit on other days can only be granted in exceptional cases, on application to the secretary, and subject to the approval of the resident medical officer in charge of the case.

VII.—Patients must in all respects conform to the regulations from time to time prescribed by the House Committee for the due government and management of the hospital and all matters incidental to their position therein and removal therefrom.

The treatment of the patients in the pay wards is undertaken by the honorary medical staff gratuitously, under the same conditions as the treatment of patients in the free wards.

### Dr. Glover's Action.

Dr. Glover, who is one of the three elected representatives of the medical profession upon the medical council, has always taken the keenest interest in the hospitals, is an active member of the council of the Metropolitan Sunday Fund, and who is deservedly and highly respected by the profession and by the inhabitants of North London, many of whom are his own patients, gave notice that he would move a resolution at the annual meeting on the 22nd ult., the effect of which would be to make all the beds in the hospital free. A copy of this resolution will be found on page 390. It will be gathered from what has already been stated that this proposal of Dr. Glover's was not only opposed to the constitution of the hospital, but that it cut at the very root of the principles upon which the institution had been built up, and in support of which the funds to erect and maintain it had been given. Mr. Burdett accordingly gave notice to the governors that he would move an amendment (see page 390), affirming that the system of pay wards now in operation should be so modified that the patients in those wards might, if they desired it, be attended by outside practitioners of their own selection. In order not to commit the hospital unduly, he further proposed that a special committee of governors should be appointed to prepare a detailed scheme to be submitted to a special meeting in June, and that such scheme should be worked experimentally for a period of five years. It will be seen that Mr. Burdett's proposals were practically identical with those contained in the original scheme circulated throughout North London, which was issued under the authority of Mr. D. Marshall Lang, the present deputy chairman of the hospital, and the then chairman of the Executive Committee, and Dr. G. W. Potter, Messrs. T. F. Black and J. H. Baldwin, the honorary secretaries, whose names were appended to it. It is interesting to note that Mr. Lang was present, but took no part in the discussion on Friday week; that Mr. Black was prevented from being present by illness; and that Dr. Potter seconded the amendment to put the scheme in force as it was originally drawn, subject to the experimental trial of five years.

### The Effect of the Amendment.

The chairman of the annual meeting seems to have forgotten these facts, as, to the surprise of many present, he declared the amendment to be a vote of censure on the committee, which the history of the amendment proves to be absurd on the face of it, and which Mr. Burdett offered to place beyond doubt by altering the wording of his amendment, so as to leave the selection of the special committee to the committee itself. We commend these facts and the report of the proceedings at the annual meeting to the candid and earnest consideration of all the governors of the Great Northern Central Hospital, and of the general practitioners who have heretofore been opposed to the present regulations for the admission of in-patients to the pay wards. It cannot be urged as a valid objection to the proposal to allow each pay patient to be attended at his option by his own medical practitioner, that such a plan would take the control of the pay wards out of the hands of the committee and make the management of the hospital impossible. We say this objection cannot be urged, for the reason that the pay wards are situated in a separate block; that access to that block can be obtained without entering the hospital proper, where the free patients and the great majority of the beds are situated; and that the whole building has been so planned as to facilitate the enforcement of adequate regulations for the treatment of pay patients in the pay wing, such regulations necessarily providing for the attendance of outside practitioners, on the same plan as that adopted at St. Thomas's Hospital, and the Home Hospital in Fitzroy Square. The effect of the rejection of both the amendment and Dr. Glover's resolution is to leave the admission of pay patients to be regulated and continued under the existing rules as set forth above.

### A Plea for Unity.

The majority of the governors present at the meeting on the 22nd ult. determined to uphold the principles embodied in the constitution which the Great Northern Central Hospital was founded to carry out. This being so, we are confident that the interests of the public, of the medical profession, of the patients, and of the governors can be reconciled and brought into line without much delay or difficulty, seeing that the facts here stated point to the way in which harmony can be secured, and afford a guarantee that everybody interested is prepared to work with the sole object of making the Great Northern Central Hospital an example and an object lesson to all the general hospitals of the country. The Great Northern Central Hospital is the only building erected for hospital purposes in England which has provided adequate accommodation for combining the free with the graduated pay system of hospital relief. It would be a reflection upon the intelligence and capacity of everybody concerned, to permit the continuance of any cause of friction in carrying out this system. We confidently hope that with the co-operation of the medical staff, the committee, the general practitioners, and the governors, the administration of the Great Northern Central Hospital may soon be placed upon the best lines. Such a result must yield a success which will cause many hospitals to reorganise their methods of relief, and to follow an example which must then commend itself to the judgment and approval of all thoughtful persons.

---

### THE PAY SYSTEM.

### THE GREAT NORTHERN CENTRAL HOSPITAL.

